# Seismological asperities from the point of view of dynamic rupture modeling: the 2007 Mw6.6 Chuetsu-Oki, Japan, earthquake

**DOI:** 10.1007/s10950-016-9569-5

**Published:** 2016-03-24

**Authors:** Hideo Aochi, Masayuki Yoshimi

**Affiliations:** 1 Bureau de Recherches Géologiques et Minières, DRP/RSV, 3 avenue Claude Guillemin, BP36009 Orléans, , Cedex 2, France; 2 Geological Survey of Japan, National Institute of Advanced Industrial Science and Technology, AIST Tsukuba Central 7, 1-1-1 Higashi, Tsukuba, Ibaraki 305-8567 Japan

**Keywords:** Fault geometry, Ground motions, Seismic hazard, Niigata Chuetsu-oki earthquake, Dynamic rupture propagation

## Abstract

We study the ground motion simulations based on three finite-source models for the 2007 Mw6.6 Niigata Chuetsu-oki, Japan, earthquake in order to discuss the performance of the input ground motion estimations for the near-field seismic hazard analysis. The three models include a kinematic source inverted from the regional accelerations, a dynamic source on a planar fault with three asperities inferred from the very-near-field ground motion particle motions, and another dynamic source model with conjugate fault segments. The ground motions are calculated for an available 3D geological model using a finite-difference method. For the comparison, we apply a goodness-of-fit score to the ground motion parameters at different stations, including the nearest one that is almost directly above the ruptured fault segments. The dynamic rupture models show good performance. We find that seismologically inferred earthquake asperities on a single fault plane can be expressed with two conjugate segments. The rupture transfer from one segment to another can generate a significant radiation; this could be interpreted as an asperity projected onto a single fault plane. This example illustrates the importance of the fault geometry that has to be taken into account when estimating the very-near-field ground motion.

## Introduction

Predicting the ground motions for any given scenario of an earthquake is an important seismological task for seismic hazard evaluation (Douglas and Aochi 2008). Nowadays, the earthquake model is kinematically constructed based on the statistical analyses of past earthquakes obtained from various inversions or synthetic earthquake scenarios dynamically simulated (e.g., Mai and Beroza 2002; Irikura and Miyake 2011; Song et al. 2013). In some research projects, there have been attempts to carry out the ground motion simulations using the dynamically simulated earthquake models directly (e.g. Olsen et al. 2009). Indeed, for two decades, dynamic rupture models have been more commonly applied to reproduce the ground motions of recent earthquakes, such as the 1992 Landers earthquake (Olsen et al. 1997; Peyrat et al. 2001; Aochi et al. 2003). Furthermore, the characteristics of the ground motion based on the dynamic rupture models are synthetically studied in terms of rupture velocity, fault geometry, heterogeneity, and so on (e.g., Oglesby and Day 2002; Aochi and Olsen 2004; Aochi and Douglas 2006; Schmedes and Archuleta 2008; Dunham and Bhat 2008). Regardless of the progress in dynamic rupture modeling, the models are difficult to produce and calibrate. In this paper, we aim to show the applicability and the utility of the dynamic rupture models for the near-field ground motion simulations applied to the 2007 Mw6.6 Chuetsu-oki, Japan, earthquake.

**Fig. 1:**
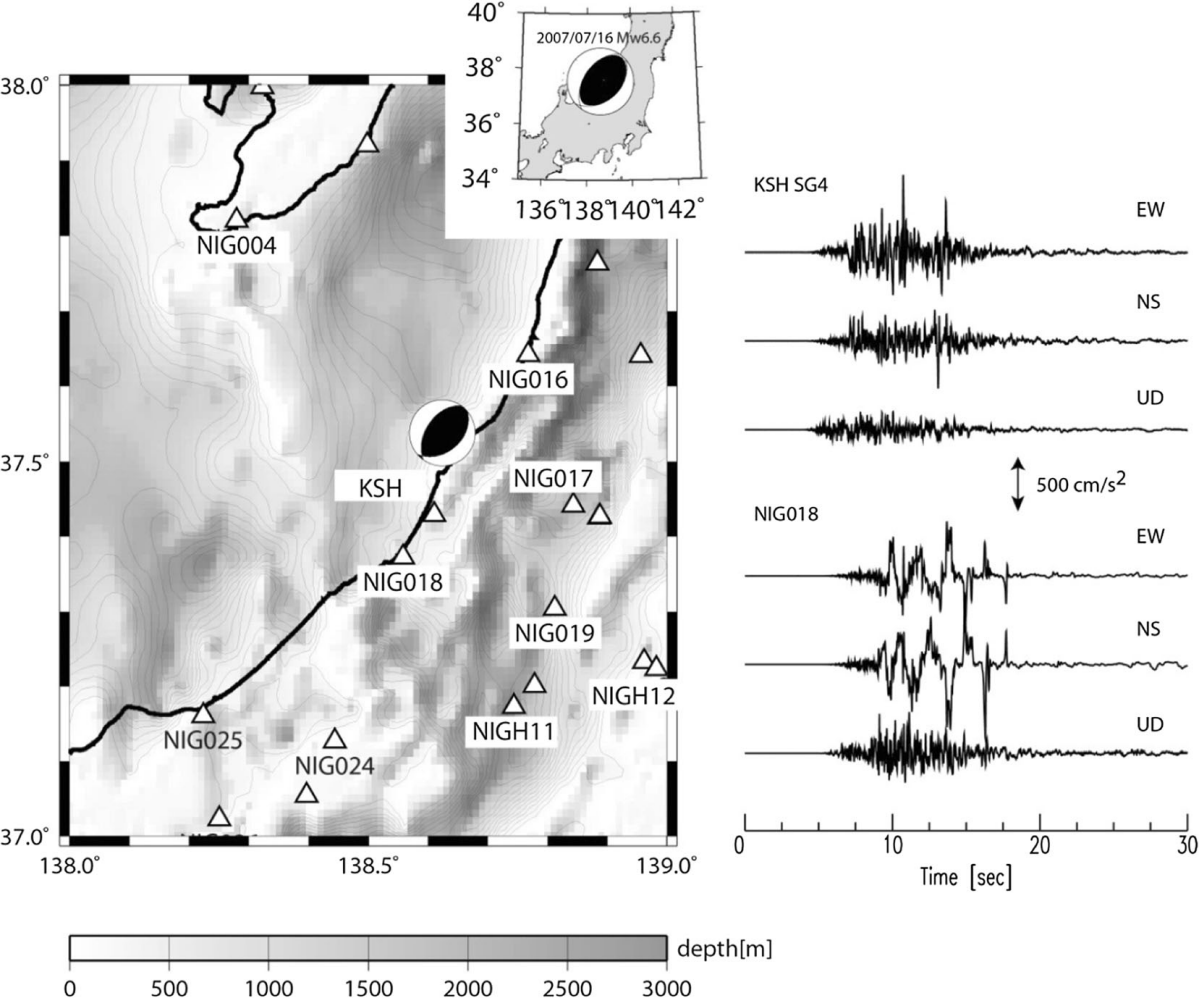
Map of the studied earthquake and area showing depth contours to the top of the Teradomari formation (Middle to Upper Miocene Mudstones), approximately corresponding to *V*_s_ = 1,000 m/s, from the 3D geological model (contours every 200 m) proposed by Sekiguchi et al. (2009). *Triangles* represent the permanent seismological observation networks, and the names are given for those which are going to be used in this study. The acceleration records are at the two nearest stations, KSH (SG4 at depth of 250 m) and NIG018. No frequency filter is applied

The 16th July 2007 Chuetsu-oki earthquake (Niigata prefecture, Japan; Fig. 1) led to some damage in Kashiwazaki and the shutdown of the Kashiwazaki-Kariwa Nuclear Power Plant (NPP), located near station KSH. The dense seismological observational networks, the geodetic observations, and the aftershock analyses in this region reveal the complexity of this earthquake in every aspect: source, propagation, and site (e.g., International Atomic Energy Agency 2007). The capability of reproducing the observed records through numerical modeling becomes a key issue for testing our knowledge and understanding of the quantitative seismic hazard assessment at any location, in particular in the very near field of the causal earthquake source area (e.g., Irikura 2010). This paper focuses on the input ground motion at the nearest seismological observations—the borehole data within the site of Kashiwazaki-Kariwa NPP (KSH–SG4). Utilizing the best available 3D geological model, we carry out the ground motion simulations based on different earthquake source models including both kinematic and dynamic descriptions. The purpose was to emphasize how kinematically inferred earthquake asperities look from the dynamic rupture models and to discuss the applicability of the dynamically simulated source models for estimating input ground motion.

## Source models of the 2007 Chuetsu-Oki earthquake

**Fig. 2:**
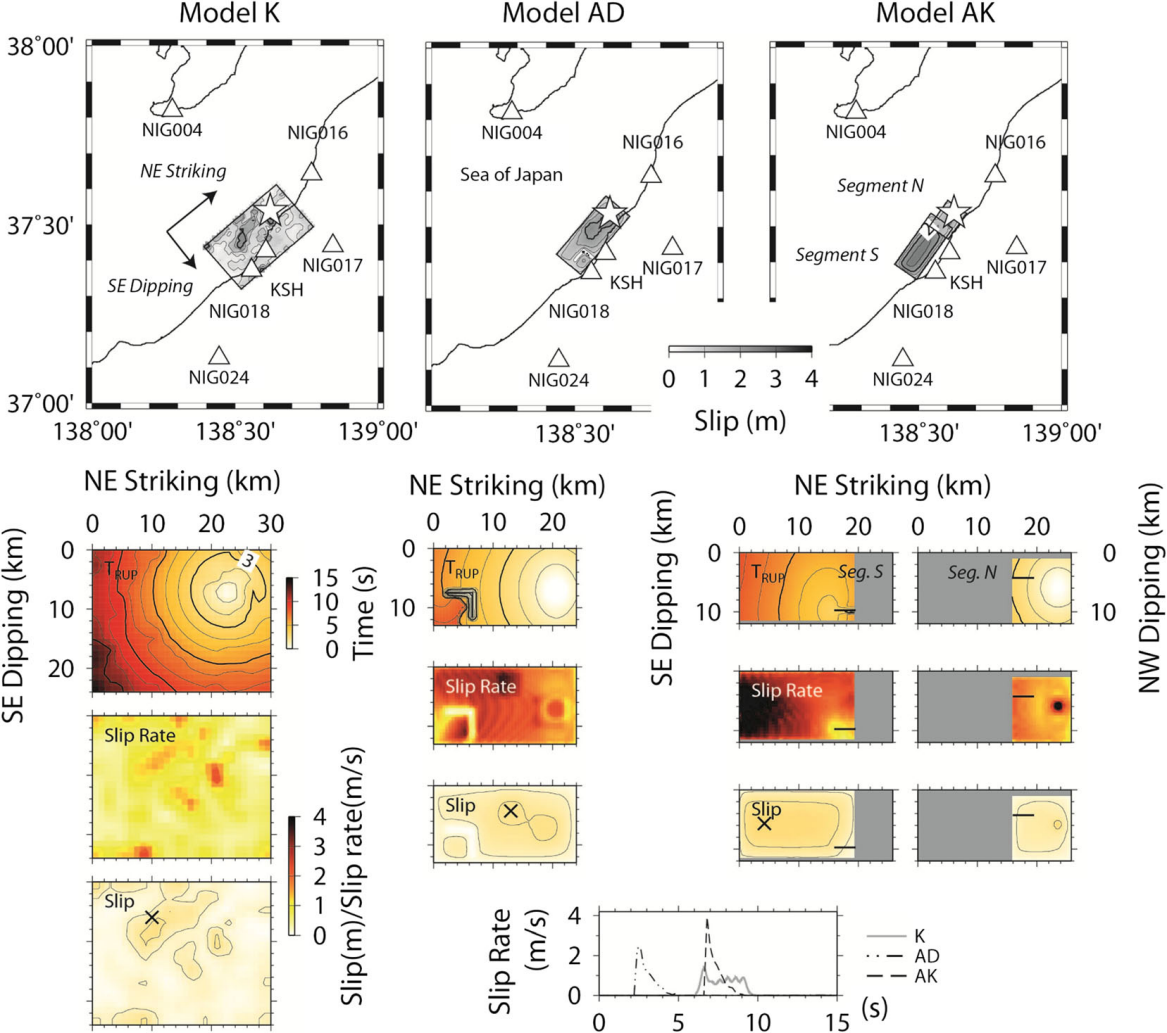
Map view showing the geometry of the three source models and the locations of stations. Model K: a kinematic source model derived from model B of Aoi et al. (2008). Model AD: a dynamic source model with three strong motion generation areas simulated in Aochi and Dupros (2011). Model AK: a dynamic source model with rupture on two cross-cutting faults (NW- and SE-dipping segments in the north and south, respectively) simulated in Aochi and Kato (2010). *Stars* represent the epicenter locations in the map. In the *bottom*, the rupture time (*T*_RUP_), maximum slip rate, and final slip distribution are shown, respectively, with the same scale. For the points of the maximum slip in the three models (denoted by a *cross*), slip rate evolutions are illustrated at the *bottom*

Figure 2 summarizes three source models for the 2007 Chuetsu-oki earthquake used in this study. Various researchers used inversion to obtain kinematic descriptions of the source process. The fault orientation (SE- or NW-dipping thrust faulting) was not evident just after the earthquake mainly because the estimated rupture zone is off the coast and the routinely determined location of the aftershocks were inaccurate (Aoi et al. 2008). The observed strong ground motion along the coastline suggested a NW-dipping fault such that the ground motion could be influenced by rupture directivity. However, the interpretation for faulting based on a SE-dipping faulting became widely accepted (e.g., Miyake et al. 2010) according to the relocation of the aftershocks from temporary observation networks (e.g., Shinohara et al. 2008; Kato et al. 2008). For this study, we adopt one of the SE-dipping fault models, model B of Aoi et al. (2008), as a reference, though we refer to it as model K (Fig. 2). This model was also used in previous ground motion simulations (Aochi et al. 2013a).

**Fig. 3:**
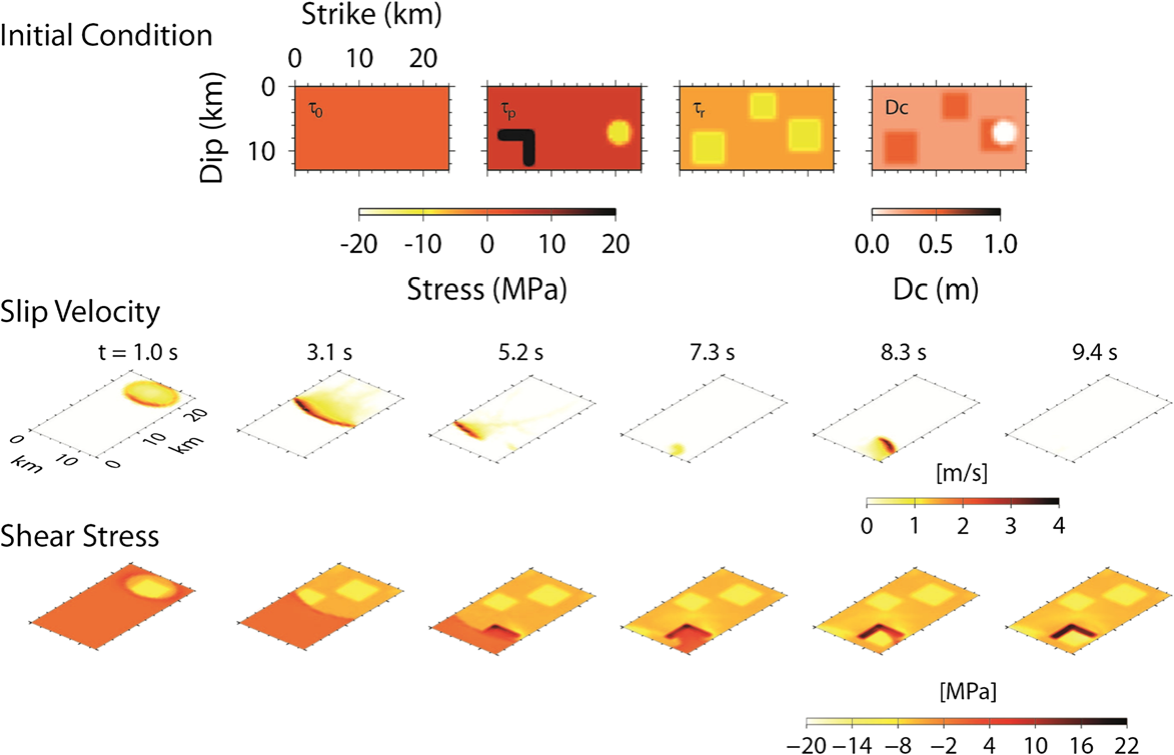
Dynamic model parameters (initial shear stress, *τ*_0_; peak strength, *τ*_p_; dynamic stress, *τ*_r_; and critical slip displacement, *D*_c_) and snapshots of slip velocity and shear stress for source model AD—dynamically simulated as rupture on a single fault plane with three asperities (modified after Aochi and Dupros 2011). Note that the slip-weakening rate, (*τ*_p_ − *τ*_r_)/*D*_c_, is the same everywhere

The strong ground motion observed in the near field (a distance from the ruptured fault plane closer than 10 km) was the subject of a major debate about the reliability of seismic hazard estimation (Strasser and Bommer 2009; Baumann and Dalguer 2014). The nearest seismic stations (KSH and NIG018; Figs. 1 and 2) are located above the inferred ruptured area along the fault strike. The ground motion at these stations is significantly affected by near-field effects of the earthquake source, such as asperity locations. They were not used in the inversion of Aoi et al. (2008). Irikura (2008) proposed a characteristic model consisting of three asperities, identified as strong motion generation areas (SMGAs). These localized asperities should produce recognizable phases in the seismograms. This characteristic earthquake model was further studied by dynamic modeling assuming a slip-weakening friction law (Aochi and Dupros 2011) and re-illustrated in Fig. 3. From the dynamic point of view, these SMGAs correspond to areas that have stress drops two times larger than the rest of the fault area. This is consistent with the generalized strong motion recipe of Irikura and Miyake (2011). Irikura (2008) proposed that the rupture of the third asperity located in the southernmost end was in the opposite direction of the general southward rupture propagation of this earthquake (multi-hypocenter model). In order to reproduce this effect dynamically, the third asperity needs to be partially surrounded by barriers so that the rupture first moves to the southern edge of the asperity and then ruptures to the northern edge (see the snapshot at 8.3 s in Fig. 3). This dynamic model is presented as model AD.

**Fig. 4:**
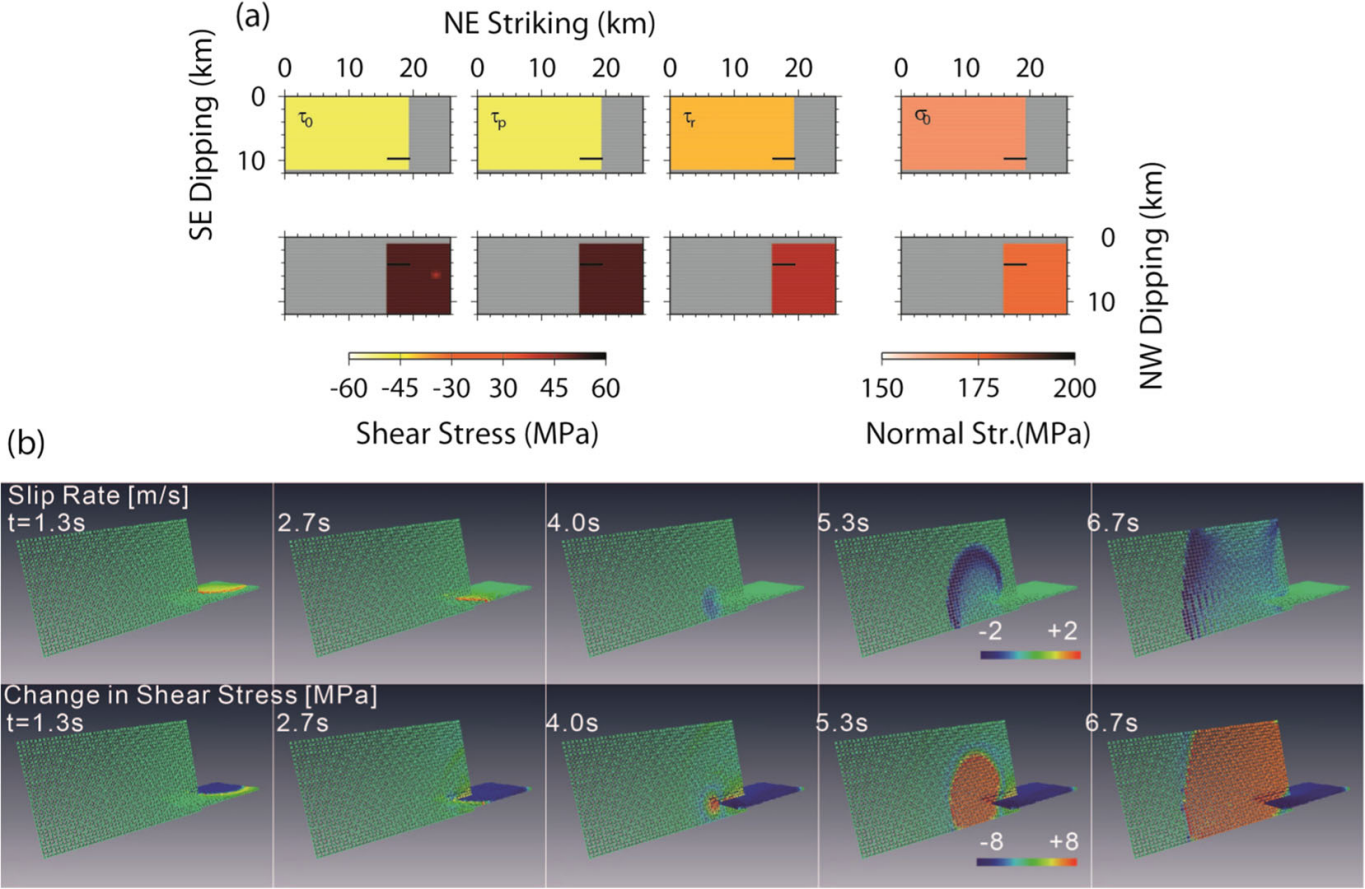
Dynamic model parameters (initial shear stress, *τ*_0_; peak strength, *τ*_p_; dynamic stress, *τ*_r_; and initial normal stress, *σ*_0_. Note that the slip-weakening rate is 30 MPa/m and is given everywhere) and snapshots of the slip rate and shear stress for the dynamic rupture process (model AK) on two conjugate fault segments (modified after Aochi and Kato 2010)

Most geodetic studies, on the other hand, propose two segmented, conjugate fault planes (Aoki et al. 2008; Nishimura et al. 2008), which were also inferred from the most accurate aftershock distributions (Shinohara et al. 2008; Kato et al. 2008). Aochi and Kato (2010) previously carried out dynamic rupture simulations for the conjugate fault geometry consisting of a northern NW-dipping segment (segment N) and a southern SE-dipping one (segment S). They varied the intersection angle and the overlapping distance, as illustrated in Fig. 4. Among their 45 simulations, only 14 show a sequential rupture transfer from one to another segment. We adopt one scenario in which the model parameters are moderate: a frictional coefficient of 0.3, an overlapping distance of two segments of 4 km, and a dipping angle of SE-dipping fault of 45°. This is designated as model AK.

## Numerical simulations of ground motion

### Simulation methods

The complexity of the 3D geological structure is well known in this region due to its complex tectonics. Aochi et al. (2013a) used the three available structure models of this region (Kato et al. 2008; Fujiwara et al. 2009; Sekiguchi et al. 2009) for ground motion simulations and discussed the validity of each model. Ground motion using the 3D structure obtained by tomography (Kato et al. 2008) shows a good coherence on rock sites that are situated close to each other. On the other hand, the models calibrated based on the geological map and the geophysical cross-sections (Fujiwara et al. 2009; Sekiguchi et al. 2009) are generally suitable for the soft sites. However, even when the models are good enough for the direct waves at certain stations, it is still difficult to obtain coherent phases for the later seismic arrivals. In this study, we adopt the 3D structure model by Sekiguchi et al. (2009) which has integrated geological layers, as shown in Fig. 1, and has a minimum shear velocity of *V*_min_ = 400 m/s.

For ground motion simulations, we use a fourth-order finite-difference method (Aochi et al. 2013a, b) with a grid spacing of *Δs* = 80 m for a dimension of 110 km (EW) × 120 km (NS) × 30 km (UD). The ground surface is approximated as flat, and the Sea of Japan is not taken into account. The maximum reliable frequency is estimated as *f*_max_ = *V*_min_/(5 ⋅ *Δs*) = 400/(5 ⋅ 80) = 1.0 Hz. The time step is 0.004 s; the calculation is run for a duration of 60 s. The simulation procedure for generating ground motion is basically the same for both the kinematic and dynamic source models, as in Aochi and Dupros (2011) and Aochi et al. (2013a), but using finer grids in this study. Any finite source can be introduced as a series of point sources with a predefined slip function of any arbitrary shape (Aochi et al. 2013b).

The included source model has been previously presented; its characteristics are summarized in Fig. 2. We propose the following models: the kinematic model K has a large rupture area, while the dynamic rupture models AD and AK have shorter rupture dimensions. The slip velocity function is also smooth (lower peak and longer duration) in model K and is shaper in AD and AK.

### Simulation results

**Fig. 5:**
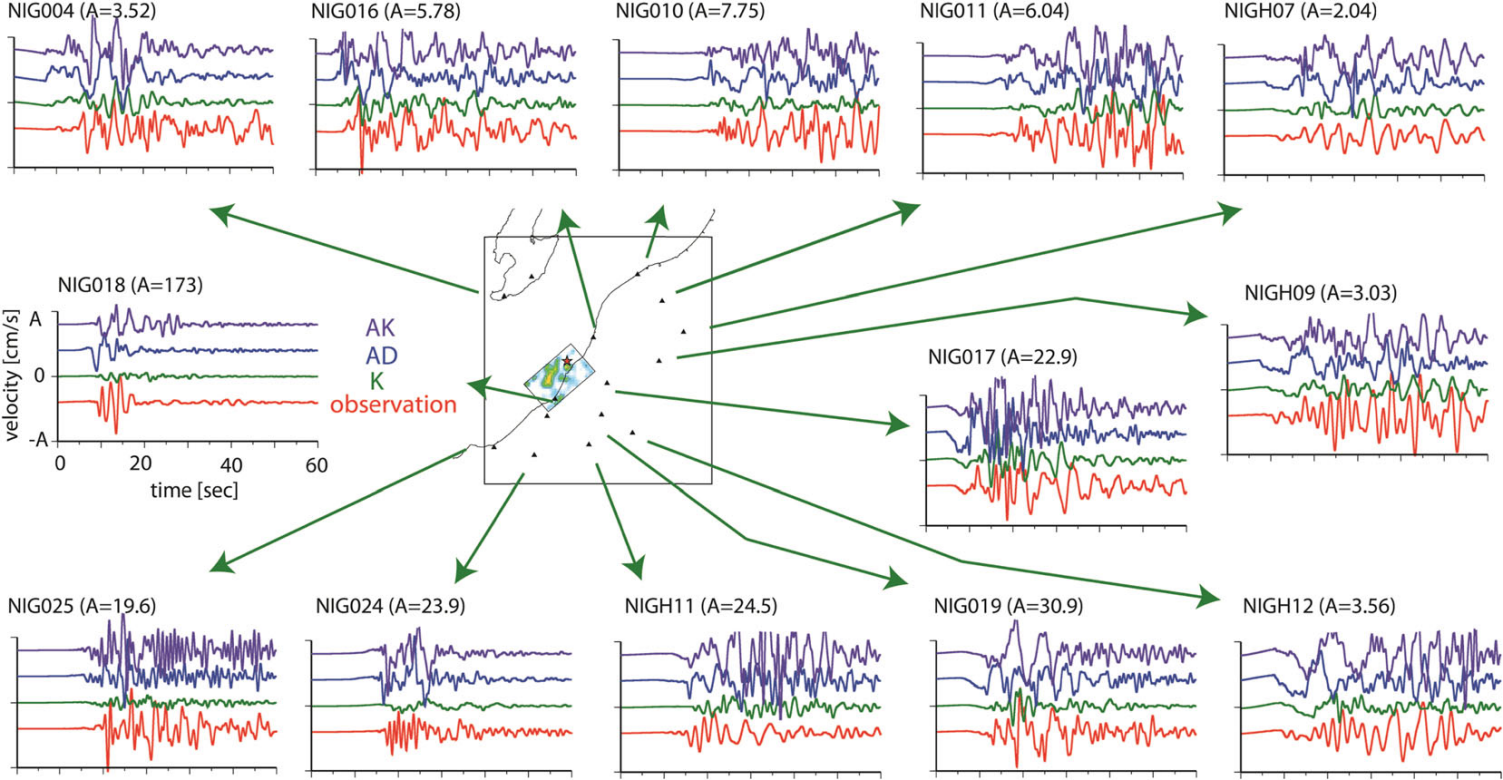
Comparison of the synthetic EW ground motion from the three source models (K, AD, and AK) with the recorded EW velocity. The ground motions were filtered between 0.1 and 1.0 Hz. For reference, the source model K is illustrated on the map

**Fig. 6:**
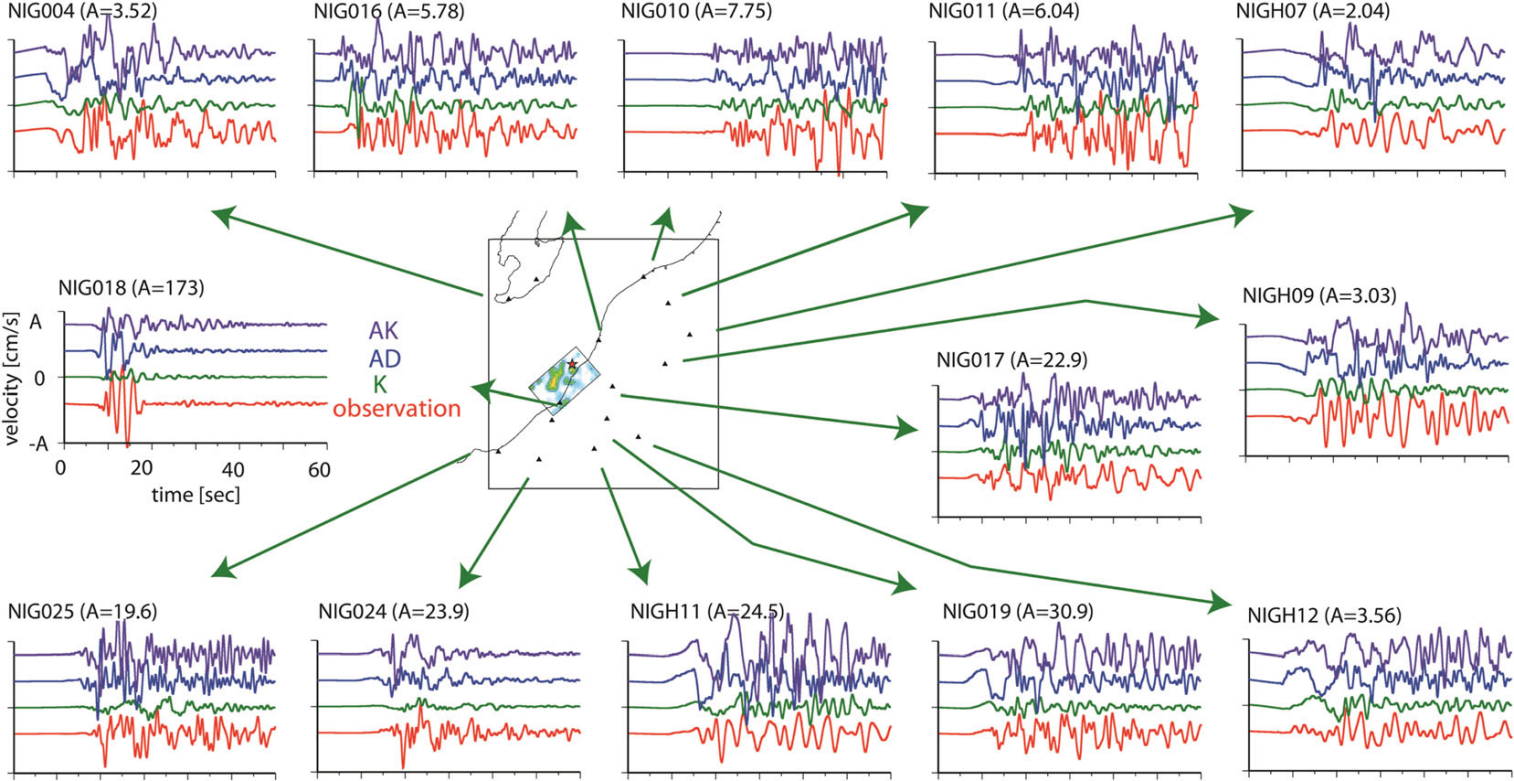
Comparison of the synthetic NS ground motion from the three source models (K, AD, and AK) with the recorded NS velocity. The ground motions were filtered between 0.1 and 1.0 Hz. For reference, the source model K is illustrated on the map

**Fig. 7:**
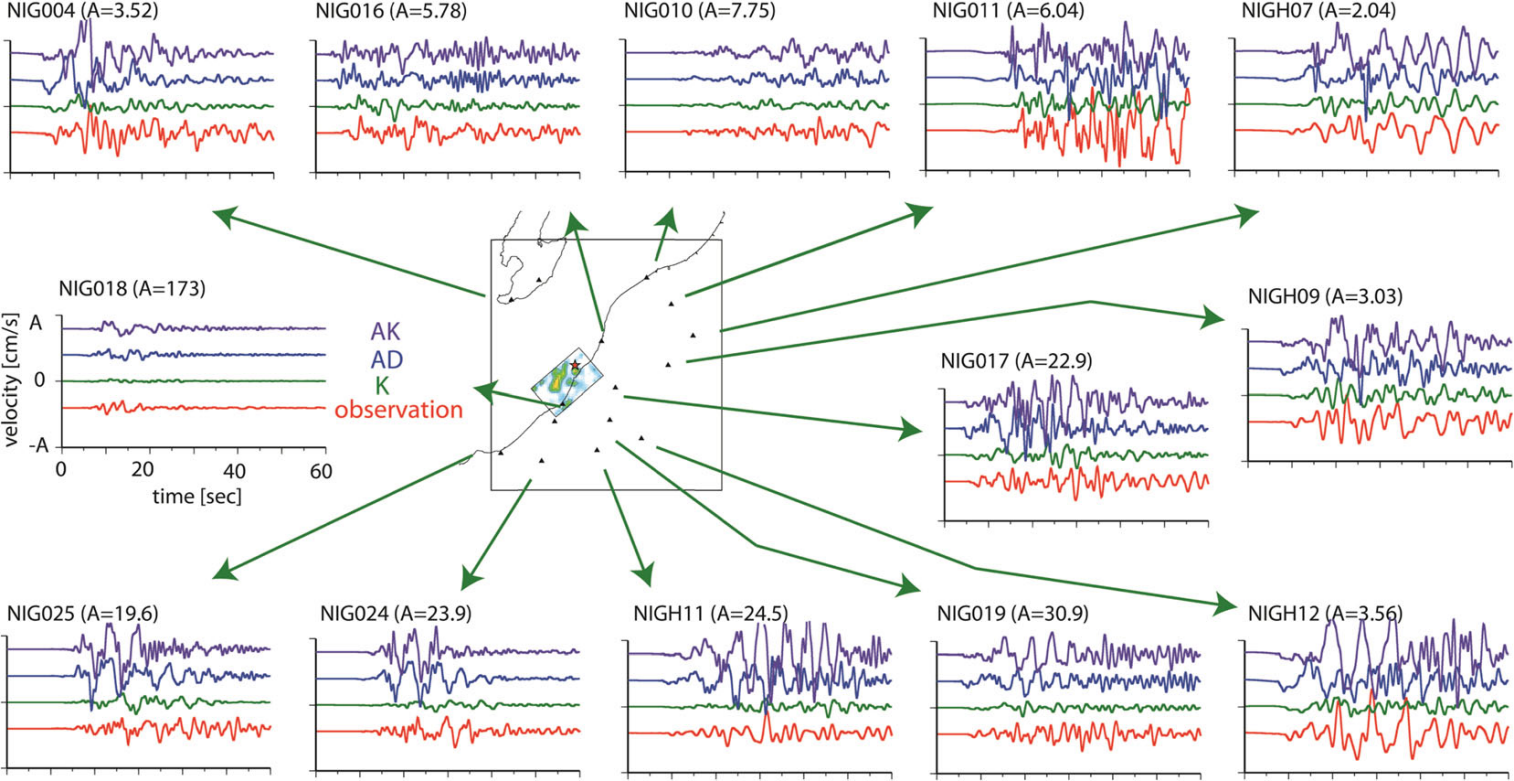
Comparison of the synthetic UD ground motion from the three source models (K, AD, and AK) with the recorded UD velocity. The ground motions were filtered between 0.1 and 1.0 Hz. For reference, the source model K is illustrated on the map

Figures 5, 6, and 7 show the simulated ground motions for the nearby stations. For model K, the synthetic ground motions have been presented by Aochi et al. (2013a). The dynamic rupture models (models AD and AK) were computed using the 3D geological model. The ground motions are aligned at the origin time of each simulation which coincides with the origin time of the hypocenter (10:13:22.16 local time). All ground motions are bandpass-filtered between 0.1 and 1.0 Hz. The kinematic model K uses a simpler 1D structure. Using only a portion (14 s around the direct S wave’s arrival) of the seismograms, the reconstruction of the full waves in a 3D structure does not always ensure the coherent fitting of the waveforms even within this frequency range. At the nearest station, NIG018, it is known that the soft soil had been subjected to liquefaction (Fig. 1) so that the synthetic ground motions cannot be compared directly to the observations.

It is difficult to capture the waveform characteristics precisely in the backward direction of the rupture propagation, namely, in the northeast area (NIG10 and NIG11). However, in the forward direction in the southwest area (NIG024 and NIG025), we find that the characteristic waveforms are better simulated by the dynamic model (AK) than by the kinematic model (K). This is an important feature. The kinematic model is based on an inversion that might have missed or underestimated significant behavior of the rupture process due to a priori constraints or smoothing of the inversion. Therefore, the source model might underestimate the strong ground motions that are closely related to the source process. The dynamic models AD and AK produce similar waveforms, in particular at NIG024, NIG025, NIG004, and NIG019, and seem to be closer to the observations than model K. The similarity of the resultant ground motions for the two models, AD and AK, indicates that the asperities inferred on a single fault plane can be represented by the geometrical irregularities of the fault system.

**Fig. 8:**
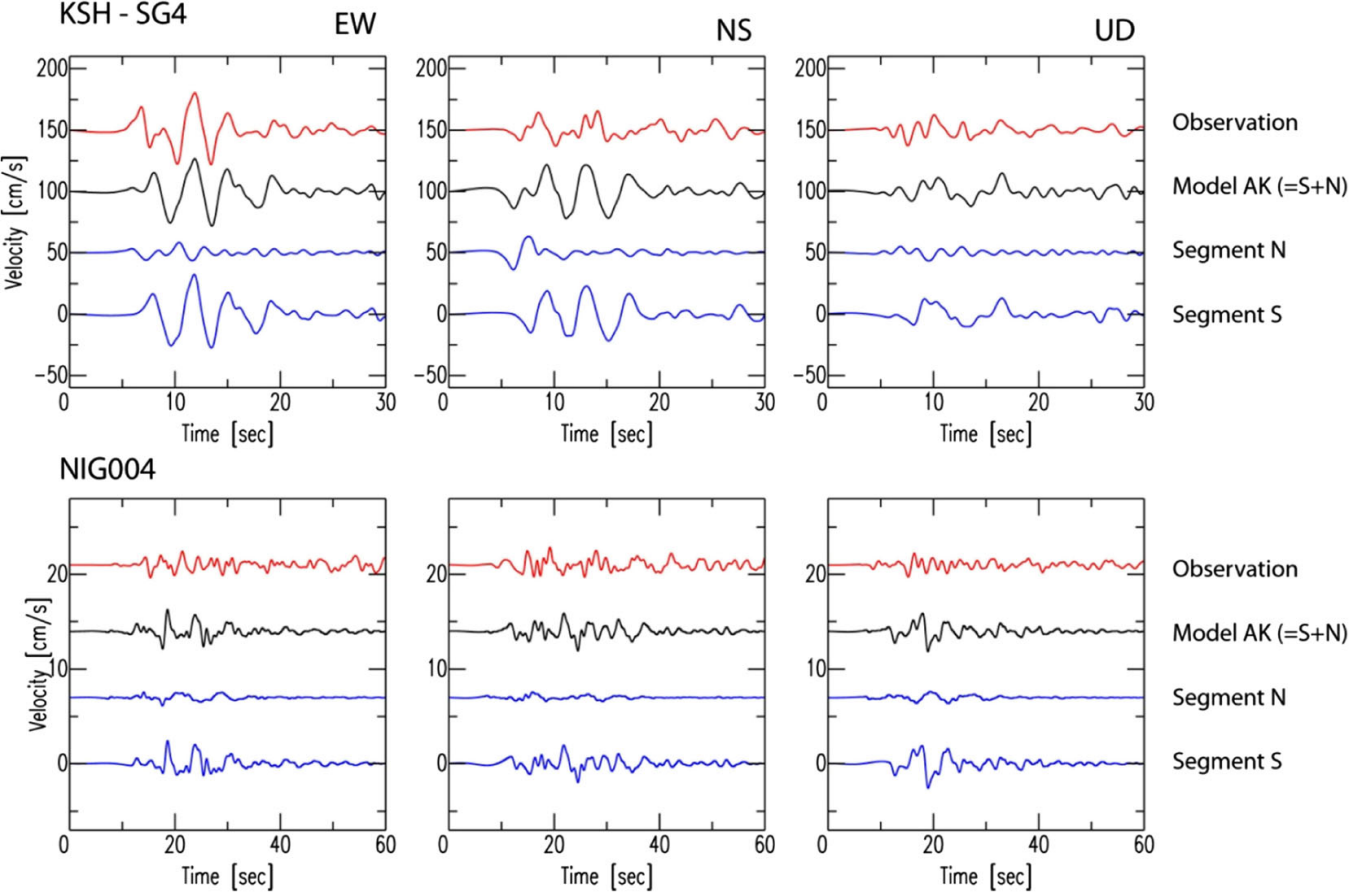
Comparison of the synthetic ground motions from either segment (segment N or S only) of model AK with the recorded velocity. The ground motions were filtered between 0.1 and 1.0 Hz. For reference, model AK (namely, the summation of segments N and S) is also shown

**Fig. 9:**
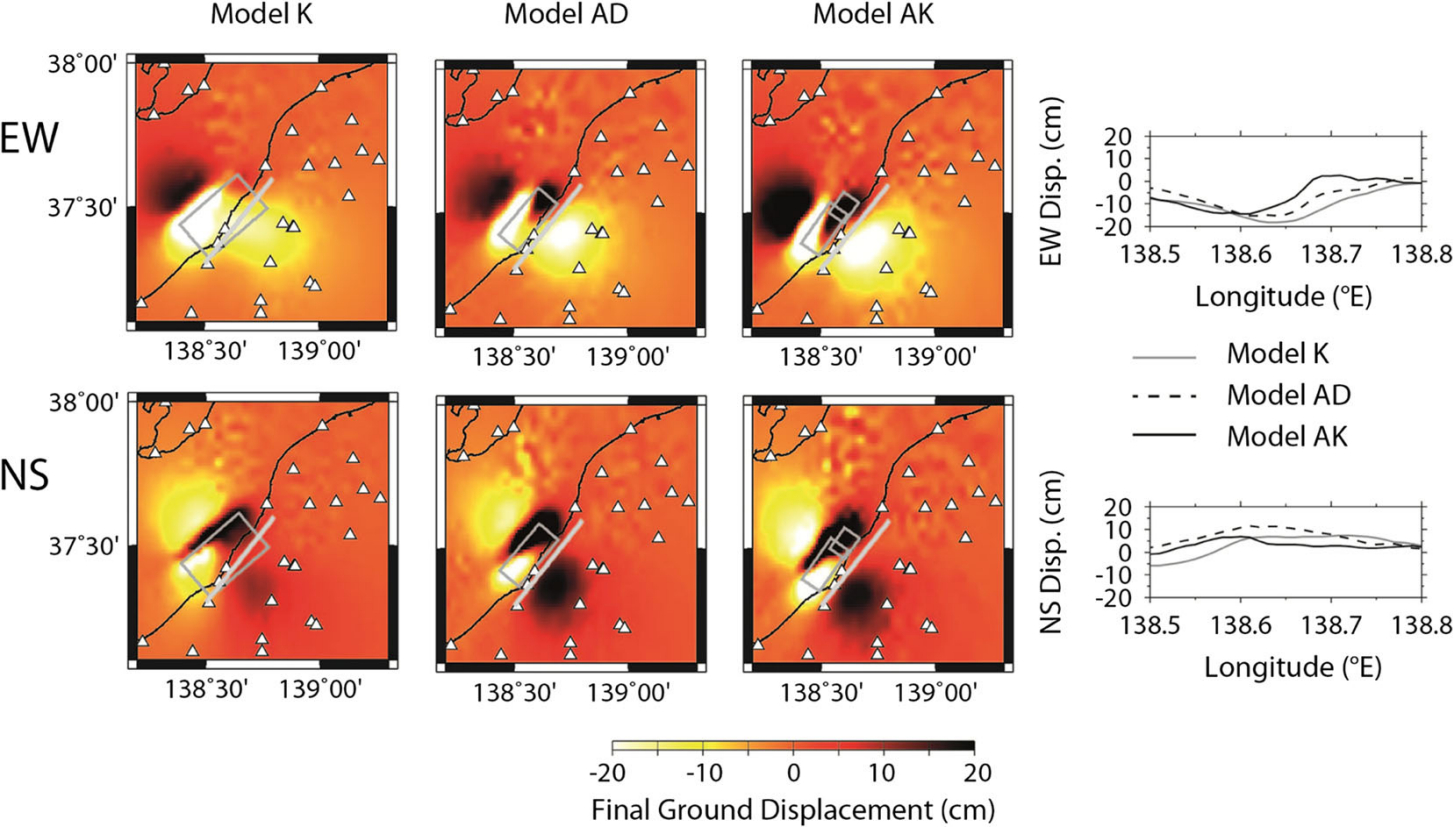
Comparison of the synthetic final ground displacement in the EW and NS components from the three source models (K, AD, and AK). The causal fault planes are projected on the map. The profiles are shown along a *white line* on the map, along the coastline

In Fig. 8, we compare two additional simulations in which we use either segment (N only or S only) of model AK to clarify the contribution of the non-planar fault structure. At the closest KSH station, the contribution is clear. The main features of the waveforms are reproduced by segment S, which is closer to KSH and releases 71 % of the total seismic energy. On the other hand, it is observed that the first pulse is represented by segment N. Such identification becomes difficult at NIG004 as the wave propagation is perturbed during a longer propagation distance. We also compare the final displacement field on the ground surface among models K, AD, and AK in Fig. 9 since two fault segments are proposed by geodetic observation (Aoki et al. 2008; Nishimura et al. 2008). A difference is hardly found in the vertical component (UD). In the horizontal components, model AK generates a clearer change in trend, although, again, the field is governed principally by segment S. The comparison between the planar models (K and AD) and the non-planar one (AK) has important implications for seismic hazard. Given that it is difficult to identify a priori any asperities on a fault plane before an earthquake, it may be more important to determine the fault structure first.

## Criteria for ground motion estimation

This paper does not aim to improve the model parameters, but tries to show the performance of different earthquake models in generating the ground motion, in particular the performance of the two dynamic rupture models. Although inversion of dynamic rupture parameters has been done for a decade (Peyrat et al. 2004; Ruiz and Madariaga 2011; Douilly et al. 2015), the number of inverted model parameters is usually limited to about 10 due to the high nonlinearity of the system and computational costs. This means that one cannot expect the same degree of spatial or temporal resolution as found in kinematic inversions unless the model parameters of dynamic rupture are constrained by kinematic inversion results (e.g., Peyrat et al. 2004). For the purpose of the ground motion prediction for seismic hazard, the engineers are more interested in the ground motion parameters than in coherent waveforms. Aochi and Douglas (2006) proposed to compare statistically the ground motion parameters at numerous points from the simulations with the ground motion prediction equations in terms of peak ground acceleration (PGA), peak ground velocity (PGV), response spectral acceleration, Arias intensity, and relative significant duration.

Olsen and Mayhew (2010) propose a goodness-of-fit (GOF) criterion for the validation of the broadband synthetics, which consists of different tests on ground motion metrics. The GOF score (normalized to 100) is defined as the complementary error function (erfc) of a normalized residual, NR (Olsen and Mayhew 2010)

1 $$ \operatorname{GOF}=100\times erfc\left(\operatorname{NR}\right),\ \mathrm{where} \operatorname {NR}=\frac{2\left|x-y\right|}{x+y} $$

where *x* and *y* are two sets of positive scalar metrics. By associating a weight on the GOF score calculated for each of the metrics, the average GOF score is obtained. Olsen and Mayhew (2010) used the metrics consisting of PGA, PGV, peak ground displacement (PGD), averaged response spectral acceleration (RS), Fourier spectrum (FS), energy duration (DUR), and cumulative energy (ENER) for broadband synthetics between 0.1 and 10 Hz for the 2008 Mw5.4 Chino Hills earthquake.

**Fig. 10:**
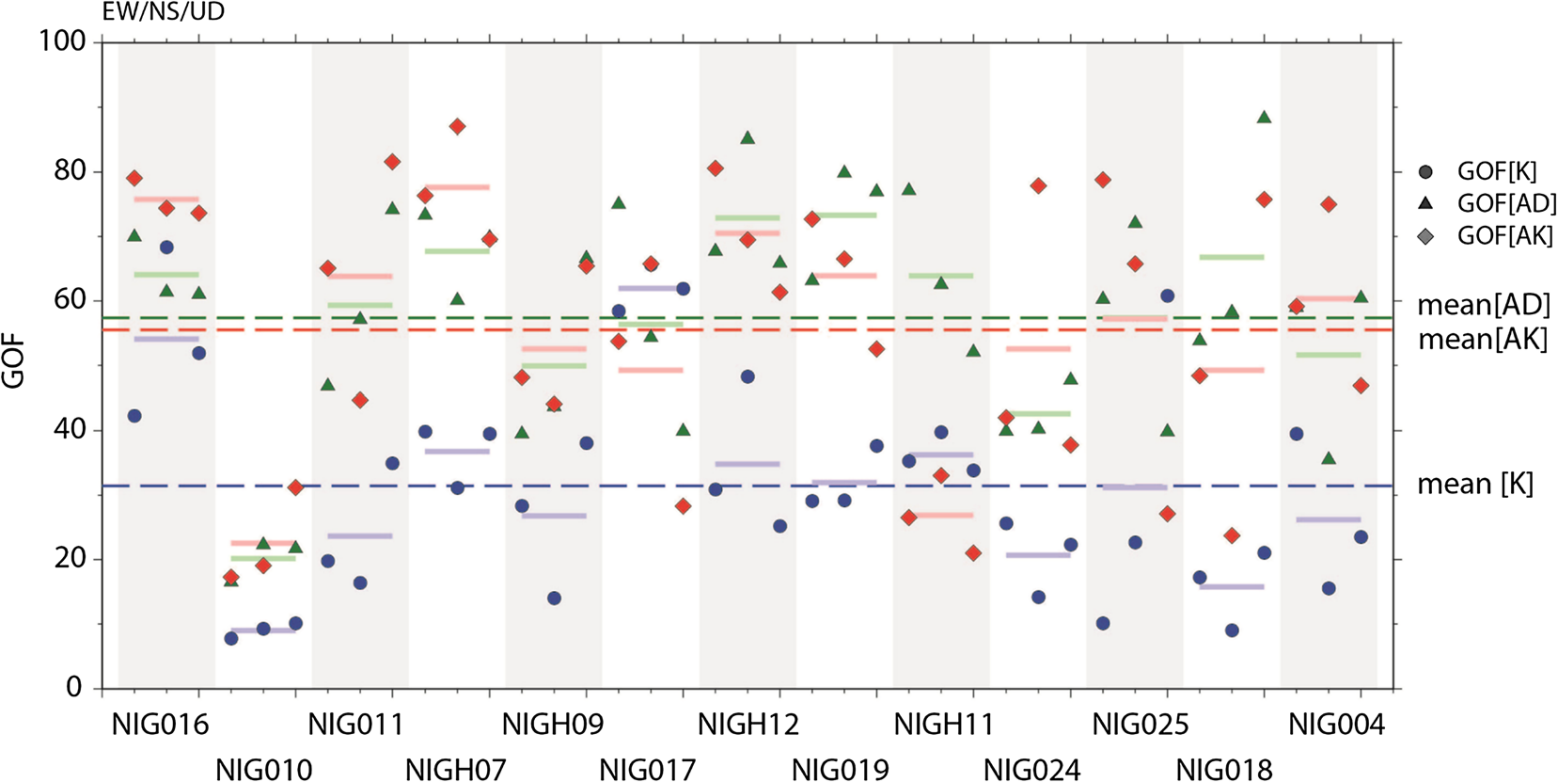
Comparison of the GOF scores for the synthetics from the three source models (K, AD, and AK) with respect to the observed seismograms. The GOF score is an average of the six metrics (PGV, PGD, DUR, RS, FS, and ENER) calculated from the waveforms filtered between 0.1 and 1.0 Hz. *Broken line* shows the average GOF score of each source model over all the stations. *Thick short line* is the GOF score averaged for each station. GOF scales are from 0 (poor) to 100 (excellent). The detailed scores by each ground motion parameter are shown in Tables A1, A2 and A3 of the Appendix

In this paper, our simulations are limited to low frequencies. We define the GOF score here as simply an equally weighted average of PGV, PGD, RS (1–10, 4–10, 2–4, and 1–2 s), FS (0.1–1, 0.1–0.25, 0.25–0.5, and 0.5–1.0 Hz), DUR, and ENER for the synthetics with respect to the observed seismograms filtered between 0.1 and 1.0 Hz, as already shown in Figs. 5, 6, and 7. Figure 10 shows the GOF scores calculated for the waveforms based on models K, AD, and AK at surrounding stations; the detailed scores are shown in Tables 1, 2, and 3 of the Appendix for each ground motion parameter to figure out the impacts in the synthetic seismograms. It is generally considered that the score is “excellent” for more than 80 %, “good” for more than 60 %, “fair” for more than 40 %, and “poor” for the rest. As observed in the waveform comparison in the previous section, the GOF scores for both AD and AK models are generally better than those from the kinematic model (K). One of the reasons is that the simulations using model K were not computed using the 3D geological model. However, we think the principal reason is that the kinematic model may have smoothed the rupture process itself because of the way in which it was originally determined by inversion, e.g., constraints on the source time function and sub-fault size. The dynamic rupture model, on the other hand, can naturally describe any drastic change in the rupture process. We also note that at particular stations, the GOF scores are not good for any source model. For example, stations NIG010 and NIG011 are located relatively far from the source in the Niigata basin, where the surface waves become dominant and continue shaking until the end of the calculation (60 s). This is one reason why the score is not good. On the other hand, we know that the nearest station, NIG018 (in Kashiwazaki basin), is at a site that liquefied; it is not possible to compare the scores when the ground motion simulation is based on a linear-elastic calculation. However, these stations are also included in the averaged GOF scores, which are still in the “fair” range for the dynamic source models AD and AK. Although the statistical analysis on the synthetics provides a means to quantify how good the results are among the different models, one should still try to understand the basic features of each seismogram.

**Fig. 11:**
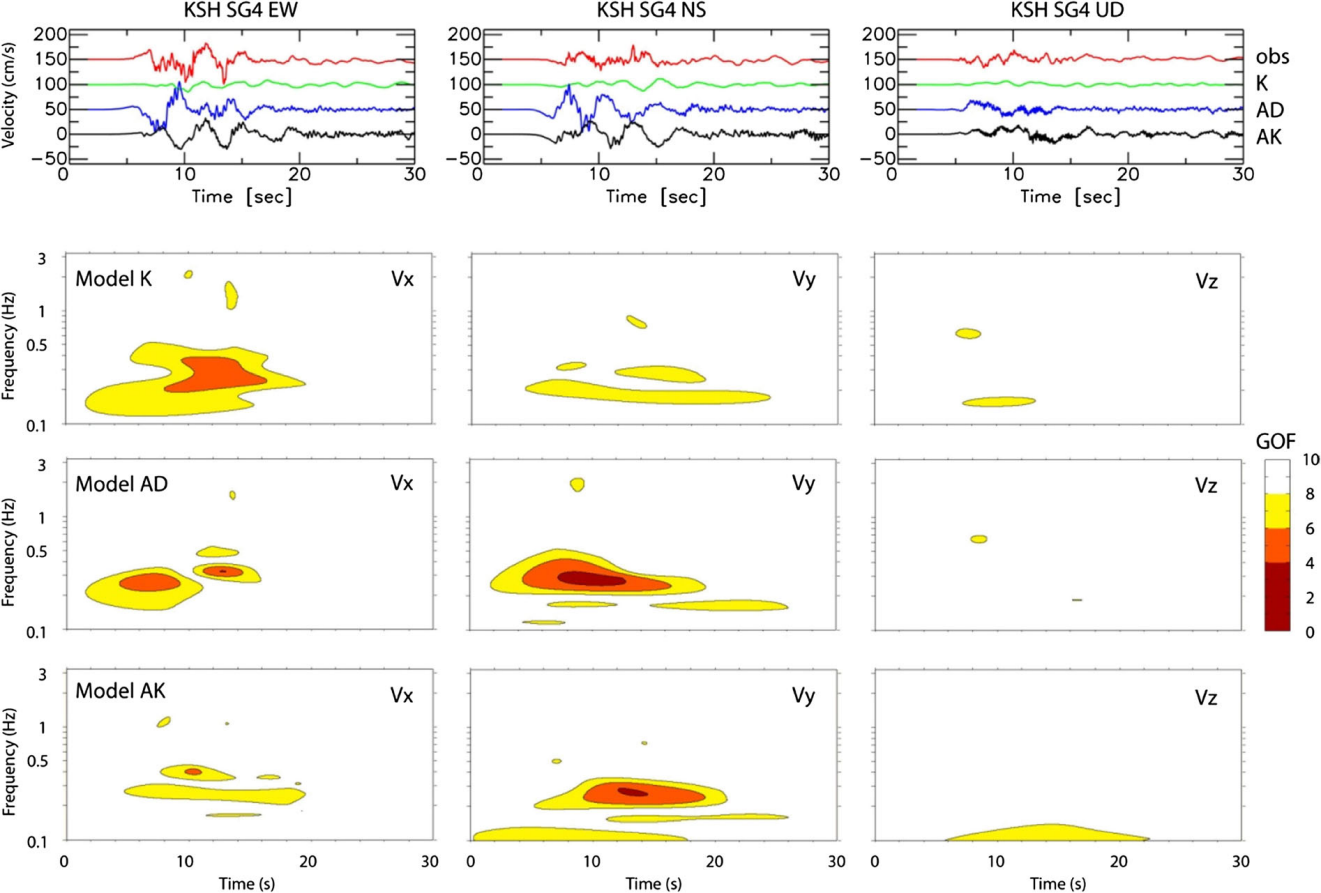
Time–frequency envelope GOF for the synthetics at SKH–SG4. The three source models are compared for three components (*x*: EW, *y*: NS, and *z*: UD) of the velocity seismograms. GOF scales are from 0 (poor) to 10 (excellent)

Kristekova et al. (2009) proposed a method to evaluate a GOF score in the time–frequency space in order to judge the local variation of the fit between the data and synthetic waveforms. Figure 11 shows the GOF score (normalized to 10) on the time–frequency envelope between the observed and synthetic ground velocities at station KSH–SG4 for all three components. Compared to the kinematic model K, the dynamic model AD for this station is slightly different for the EW component, but quite different for the NS component. This figure confirms that the dynamic model AK shows similar scores over the time–frequency range. The colored (yellow + orange) area of model AK is slightly smaller than in model K (by 23 % for the EW component). This comparison of models shows that the dynamic model can generate input ground motion equally well as the kinematic model. This suggests that a hybrid approach, i.e., combining a low-frequency dynamic rupture with any stochastic approach, could be used for generating the broadband ground motion in future applications, as suggested with various finite-source models (e.g., Graves and Pitarka 2015; Crempien and Archuleta 2015; Olsen and Takedatsu 2015).

## Discussion and summary

The aim of this paper was to examine the performance of the dynamically simulated earthquake ruptures in computing ground motion for the purpose of quantitative seismic hazard analysis. For the 2007 Mw6.6 Chuetsu-oki, Japan, earthquake, we compute ground motion in a 3D geological model using a kinematic source model and two dynamic source models. To quantify the performance of the models, we apply a GOF criterion to the simulated ground motions. From the comparisons, we find that the kinematic model does not always have the best performance in reproducing the characteristics of the strong ground motion. In particular, it does not work well in the very near field and in the forward direction of the rupture propagation. This is probably because an abrupt change in the rupture process (rupture onset and changes in rupture velocity) may not be well simulated by the kinematic description. Two dynamic rupture models produce a similar ground motion radiation. However, the model with the asperities distributed on a single fault plane might be a projection of the rupture process on conjugate fault segments. The rupture process changes the wave radiation naturally due to the geometrical irregularities. From this perspective, the dynamic rupture model on a complex fault geometry produces a reasonable rupture scenario and wave radiation for practical applications.

For the 2007 Chuetsu-oki earthquake, the conjugate segments might be a reasonable causal source model, as inferred from the geodetic analyses (Nishimura et al. 2008; Aoki et al. 2008). Although the model parameters could be calibrated better, the rupture process on each segment could be very simple, represented by three phases: first, rupture on the NW-dipping segment; second, a dynamic rupture transfer between conjugate segments (which changes the wave radiation); and third, rupture on the SE-dipping segment. These features correspond to the three asperities commonly found from the seismological finite-source inversions. It is not possible to distinguish between the two rupture scenarios. To allow for possible mechanisms, one has to consider the possibility of rupture transfer from one segment to another and be included in probable rupture scenarios used to estimate the ground motion. Indeed, recent improvements of the geophysical observations often reveal complex fault geometries even for moderate-magnitude earthquakes, such as the 2009 Mw6.4 Suruga Bay, Japan (Aoi et al. 2010), and the Mw6.9 Iwate-Miyagi Nairiku, Japan, earthquakes (Fukuyama, 2015). In the seismological analyses, these earthquakes were mostly studied as ruptures on a single fault plane, an approximation good enough for regional or teleseismic scales.

There remains a scientific debate on the different aspects of the dynamic rupture process because the dynamics are technically difficult to solve and the frictional component of the faulting is difficult to study. However, progress over the last two decades helps us to understand various aspects of the dynamics of the earthquake mechanism. Dynamic rupture scenarios have been used to compute the ground motions for high-seismic-hazard areas such as California (e.g., Olsen et al. 2009), Japan (Sekiguchi and Kase 2012), and Turkey (Aochi and Ulrich 2015). The parameter studies on dynamic rupture allow retrieving probable rupture scenarios and ground motions (e.g., Aochi et al. 2006; Aochi and Ulrich 2015). Moreover, these dynamic simulations provide insight about the variability of the phenomena and extreme ground motions (e.g., Andrews et al. 2007).

## Appendix

Tables 1, 2, and 3 summarize the GOF scores for each ground motion parameter by component for the three source models (K, AD, and AK), respectively. The average of the scores by each seismogram is shown in Fig. 10. *DUR* energy duration, *PGV* peak ground velocity, *PGD* peak ground displacement, *RS* response spectral acceleration (averaged for periods between 1 and 10 s (the entire period of study), 4 and 10, 2 and 4, and 1 and 2 s, respectively), *FS* Fourier spectrum (smoothed to reduce variance for frequencies between 0.1 and 1 Hz (the studied entire frequency), 0.1 and 0.25, 0.25 and 0.5, and 0.5 and 1 Hz, respectively), *ENER* cumulative energy (Olsen and Mayhew 2010). For some stations, we observe that the ground oscillation does not cease yet in Figs. 5, 6, and 7 by the end of the calculation (60 s). In such cases, we had to cut the end of DUR automatically by the end of the calculation. This may be one of the reasons why the DUR score is not high compared to the other parameters.

**Table 1:** GOF scores for the defined 13 ground motion parameters by each component of the seismograms between observation and synthetics based on source model K

	DUR	PGV	PGD	RS (s)				FS (Hz)				ENER	Average
				1–10	4–10	2–4	1–2	0.1–1	0.1–0.25	0.25–0.5	0.5–1		
EW													
NIG016	33.7	35.9	48.6	49.5	54.5	51.7	27.0	30.9	54.8	42.9	24.8	10.5	38.7
NIG010	33.8	2.2	1.7	3.0	2.8	2.9	7.6	3.3	2.1	2.2	6.3	0.6	5.7
NIG011	14.8	27.7	19.7	28.5	33.7	26.7	9.1	7.7	19.9	20.2	3.4	2.0	17.8
NIGH07	49.4	54.5	41.3	37.0	26.1	69.9	24.7	29.3	27.1	58.9	18.2	7.1	37.0
NIGH09	67.0	7.9	23.4	15.1	28.9	8.3	2.0	4.3	51.7	9.5	2.1	1.5	18.5
NIG017	30.9	87.7	40.6	71.6	56.0	71.5	44.0	69.9	49.8	57.1	47.1	36.4	55.2
NIGH12	77.0	31.8	29.6	14.4	14.3	13.9	15.6	15.0	17.3	14.1	16.2	2.2	21.8
NIG019	42.8	31.5	21.5	28.7	19.0	53.7	40.8	30.6	19.0	45.3	28.3	4.4	30.5
NIGH11	48.1	16.9	30.5	38.1	41.6	43.7	20.9	34.6	43.0	55.3	29.4	11.5	34.5
NIG024	43.5	8.5	29.9	19.0	31.6	12.3	4.0	5.2	47.2	14.5	3.3	2.3	18.4
NIG025	29.3	5.1	6.3	6.5	6.9	6.3	6.1	5.8	7.4	4.2	6.6	0.9	7.6
NIG018	16.6	5.6	7.0	6.0	6.5	6.0	4.2	10.0	57.8	6.6	12.1	1.1	11.6
NIG004	7.9	76.2	67.4	45.7	38.6	67.1	21.9	21.3	18.2	52.3	10.8	7.6	36.2
Average	38.1	30.1	28.3	27.9	27.7	33.4	17.5	20.6	31.9	29.5	16.0	6.8	25.7
NS													
NIG016	38.5	88.7	64.8	99.7	92.3	81.6	95.0	59.1	59.0	82.2	51.0	30.3	70.2
NIG010	27.9	7.4	3.2	5.9	5.0	7.9	9.0	6.1	4.8	5.4	7.7	0.7	7.6
NIG011	28.1	24.7	10.1	14.1	10.5	35.1	10.5	12.6	8.6	23.2	10.6	1.5	15.8
NIGH07	52.3	42.3	10.9	33.0	29.7	38.8	32.2	24.3	23.5	38.3	19.4	3.6	29.0
NIGH09	33.7	12.4	8.4	11.6	10.8	19.7	4.2	4.9	12.9	14.3	2.7	1.3	11.4
NIG017	43.2	93.1	65.4	68.2	51.5	98.7	88.8	85.7	38.4	92.6	99.7	28.1	71.1
NIGH12	78.7	63.2	46.9	45.8	53.0	36.8	36.9	29.6	25.5	42.3	26.9	7.4	41.1
NIG019	75.1	27.2	15.9	23.2	17.5	37.6	31.8	21.4	12.2	27.2	21.9	2.8	26.1
NIGH11	65.4	29.3	43.9	39.2	35.9	43.7	42.1	34.0	26.7	46.2	32.4	8.1	37.2
NIG024	44.5	6.5	8.4	7.7	8.1	6.3	8.9	7.3	10.3	5.9	7.9	1.1	10.2
NIG025	27.3	19.7	29.1	27.9	42.8	7.2	9.9	8.3	23.3	4.3	8.7	2.5	17.6
NIG018	12.2	4.5	4.2	4.4	4.7	3.9	5.1	5.7	22.9	4.0	8.2	0.8	6.7
NIG004	36.7	13.9	5.8	15.2	11.7	36.1	6.1	8.7	12.8	30.7	4.0	1.7	15.3
Average	43.3	33.3	24.4	30.5	28.7	34.9	29.3	23.7	21.6	32.0	23.2	6.9	27.6
UD													
NIG016	33.4	72.7	19.8	64.0	61.9	72.4	61.4	51.3	70.3	40.4	56.5	14.4	51.5
NIG010	27.3	7.2	6.1	7.3	5.7	12.7	17.7	8.6	3.9	6.8	16.9	0.8	10.1
NIG011	35.1	50.4	31.8	39.8	39.6	36.5	50.4	21.6	30.4	23.7	22.4	4.3	32.2
NIGH07	37.9	45.8	25.2	43.2	42.0	59.4	17.0	35.2	49.4	68.5	18.4	11.1	37.8
NIGH09	49.7	43.4	33.6	35.6	33.5	55.0	12.2	17.1	48.5	50.8	7.6	7.1	32.8
NIG017	43.4	64.4	82.4	89.4	74.6	41.9	51.0	47.1	44.6	32.1	45.8	24.6	53.4
NIGH12	91.6	10.3	8.1	10.0	8.4	16.9	8.4	14.9	16.1	21.8	12.4	1.7	18.4
NIG019	60.4	37.3	49.0	28.7	28.9	31.9	21.9	21.7	28.5	30.1	19.0	4.2	30.1
NIGH11	35.4	26.5	30.3	30.1	32.2	32.1	18.5	18.6	61.9	20.0	15.7	5.3	27.2
NIG024	25.4	20.0	35.1	19.0	20.3	20.3	8.1	11.2	22.8	24.1	6.9	3.7	18.1
NIG025	22.5	68.1	90.4	58.6	75.9	29.5	31.1	25.9	99.1	16.8	22.7	15.8	46.4
NIG018	72.0	8.6	7.9	10.7	10.3	9.0	19.0	18.9	8.0	11.6	29.8	1.8	17.3
NIG004	43.6	15.9	21.4	22.8	29.1	15.3	16.4	16.9	20.0	27.6	13.4	2.6	20.4
Average	44.4	36.2	33.9	35.3	35.6	33.3	25.6	23.8	38.7	28.8	22.1	7.5	30.4

**Table 2:** GOF scores for the defined 13 ground motion parameters by each component of the seismograms between observation and synthetics based on source model AD

	DUR	PGV	PGD	RS (s)				FS (Hz)				ENER	Average
				1–10	4–10	2–4	1–2	0.1–1	0.1–0.25	0.25–0.5	0.5–1		
EW													
NIG016	39.7	66.8	86.7	78.8	97.9	41.1	31.9	53.7	94.0	34.7	55.4	34.7	59.6
NIG010	43.4	12.8	5.4	12.0	13.6	4.1	54.0	18.2	7.5	3.0	57.2	1.2	19.4
NIG011	26.4	94.4	40.4	50.2	60.1	27.1	52.8	30.9	38.8	16.8	38.4	5.3	40.1
NIGH07	35.6	58.1	91.7	70.3	69.3	86.0	53.6	92.3	92.0	71.1	72.8	61.4	71.2
NIGH09	57.9	25.7	40.5	29.8	52.1	12.9	7.9	10.6	72.3	9.8	8.0	3.0	27.5
NIG017	54.0	78.1	71.0	82.3	77.0	99.8	88.9	82.1	82.6	93.9	67.5	92.3	80.8
NIGH12	76.1	53.0	91.7	62.6	73.0	56.8	24.4	44.9	78.0	33.9	39.8	43.7	56.5
NIG019	82.1	44.6	50.2	67.1	81.6	38.8	50.5	61.6	73.3	27.3	68.7	41.8	57.3
NIGH11	54.6	85.9	85.9	83.4	61.8	90.9	66.6	98.5	54.8	94.9	92.2	89.2	79.9
NIG024	22.6	37.8	29.4	52.8	34.5	80.0	90.6	79.5	17.1	98.7	64.3	59.7	55.6
NIG025	44.8	87.7	71.2	71.8	72.6	71.5	70.6	59.2	27.2	43.6	70.1	20.6	59.2
NIG018	56.6	57.2	75.6	71.6	99.1	49.0	25.0	50.5	11.4	26.6	53.7	28.4	50.4
NIG004	7.3	80.4	43.1	96.9	59.9	17.6	28.5	29.6	96.6	14.8	26.0	72.9	47.8
Average	46.2	60.2	60.2	63.8	65.6	52.0	49.6	54.7	57.4	43.8	54.9	42.6	54.3
NS													
NIG016	46.3	45.9	56.5	66.7	80.8	44.4	51.0	57.2	95.6	26.3	66.6	30.2	55.6
NIG010	30.1	36.8	13.3	20.1	16.9	23.2	47.9	20.5	13.0	10.9	32.9	1.6	22.3
NIG011	32.1	97.6	59.0	59.5	55.6	61.0	78.6	62.4	32.1	25.9	89.6	10.4	55.3
NIGH07	35.1	53.0	41.8	96.0	94.4	62.8	39.2	96.9	38.0	37.7	58.4	24.6	56.5
NIGH09	31.2	76.7	30.2	43.1	55.7	26.0	32.5	20.4	60.8	17.1	19.3	4.6	34.8
NIG017	37.5	35.0	57.9	54.9	63.2	53.8	23.4	42.4	98.6	97.9	22.5	60.5	54.0
NIGH12	85.0	96.2	91.1	94.3	81.6	50.4	69.1	74.0	70.3	46.0	80.7	64.1	75.2
NIG019	90.1	72.1	58.9	85.6	79.1	97.1	97.3	89.5	83.0	84.2	91.0	94.8	85.2
NIGH11	49.3	88.7	34.7	58.9	39.2	82.7	98.5	99.8	43.7	61.9	93.4	65.8	68.1
NIG024	35.8	32.5	31.3	40.4	46.0	24.8	41.7	36.4	64.7	30.4	35.8	13.0	36.1
NIG025	33.9	69.2	82.1	88.6	98.1	85.4	60.4	92.6	66.1	70.9	75.7	72.6	74.6
NIG018	65.8	66.3	50.5	64.5	70.2	50.5	90.5	81.3	21.1	39.4	86.0	33.8	60.0
NIG004	29.9	42.9	27.9	42.9	48.9	33.2	29.2	25.9	43.3	22.9	24.8	9.7	31.8
Average	46.3	62.5	48.9	62.7	63.8	53.5	58.4	61.5	56.2	43.9	59.8	37.4	54.6
UD													
NIG016	45.8	84.2	27.3	69.4	61.5	60.0	71.6	87.8	51.8	45.2	56.5	49.7	59.2
NIG010	28.3	24.8	15.1	17.0	13.9	14.8	82.9	37.7	7.3	11.0	93.9	1.7	29.0
NIG011	28.1	95.3	67.0	99.3	97.0	74.1	43.1	91.5	63.8	42.6	77.0	47.4	68.9
NIGH07	28.9	41.2	81.9	92.6	99.8	89.7	59.9	86.1	88.3	82.8	66.1	61.3	73.2
NIGH09	35.1	73.6	61.6	78.0	89.5	56.8	59.7	52.8	98.9	45.9	50.3	29.3	61.0
NIG017	43.3	50.3	34.3	36.6	18.5	93.6	51.0	62.0	12.7	52.9	44.9	48.3	45.7
NIGH12	86.6	40.9	40.3	56.1	62.9	33.5	67.9	75.4	96.0	45.9	88.3	31.2	60.4
NIG019	76.9	79.6	77.8	94.8	75.0	57.3	98.5	96.7	36.2	33.7	86.3	95.4	75.7
NIGH11	51.8	63.2	46.8	47.3	37.8	72.0	69.7	77.2	26.3	98.9	77.1	32.0	58.4
NIG024	14.0	43.7	33.1	54.7	47.9	80.2	82.3	99.3	41.4	89.7	87.4	26.0	58.3
NIG025	24.9	28.3	18.2	51.5	37.9	94.0	84.1	94.2	21.9	71.5	81.0	33.9	53.5
NIG018	96.9	80.2	99.0	86.7	73.1	78.3	95.7	84.6	81.8	63.6	61.1	93.7	82.9
NIG004	20.9	88.9	45.5	73.5	42.6	45.6	65.3	70.9	63.0	48.4	69.8	97.8	61.0
Average	44.7	61.1	49.8	66.0	58.3	65.4	71.7	78.2	53.0	56.3	72.3	49.8	60.5

**Table 3:** GOF scores for the defined 13 ground motion parameters by each component of the seismograms between observation and synthetics based on source model AK

	DUR	PGV	PGD	RS (s)				FS (Hz)				ENER	Average
				1–10	4–10	2–4	1–2	0.1–1	0.1–0.25	0.25–0.5	0.5–1		
EW													
NIG016	44.5	86.6	78.2	90.0	73.8	73.6	81.8	84.3	91.0	78.8	84.7	79.6	78.9
NIG010	26.8	17.9	10.0	17.2	16.6	11.2	62.1	23.1	8.5	6.3	61.1	1.2	21.9
NIG011	15.6	89.4	95.3	88.0	92.9	44.2	58.8	40.0	62.4	32.8	39.7	17.5	56.4
NIGH07	53.1	94.7	72.6	74.3	57.9	82.0	77.5	90.4	73.0	87.5	82.2	98.5	78.6
NIGH09	53.7	34.4	53.5	37.4	59.2	26.7	7.0	15.2	95.2	24.4	8.5	6.8	35.2
NIG017	36.5	57.1	43.3	55.1	55.7	44.5	72.8	49.7	80.7	44.6	44.6	45.9	52.5
NIGH12	77.3	72.3	99.9	87.6	89.6	91.0	70.4	91.9	54.5	68.7	79.9	76.9	80.0
NIG019	74.7	79.2	70.2	88.9	63.6	47.2	38.4	72.0	51.2	52.9	66.3	90.3	66.2
NIGH11	33.1	27.5	26.6	21.3	14.0	39.8	37.6	39.9	10.8	42.8	43.3	7.4	28.7
NIG024	17.4	42.1	32.9	51.5	37.1	66.9	95.6	89.2	18.7	69.4	70.0	48.5	53.3
NIG025	42.2	98.0	92.9	96.4	86.5	99.5	79.0	97.2	46.0	51.6	76.0	90.4	79.6
NIG018	23.9	55.1	52.0	72.3	92.6	60.1	34.5	76.5	10.8	51.6	80.5	51.9	55.1
NIG004	10.6	58.8	42.6	67.3	59.0	94.2	58.2	96.4	79.5	82.2	86.0	92.0	68.9
Average	39.2	62.5	59.2	65.2	61.4	60.1	59.5	66.6	52.5	53.4	63.3	54.4	58.1
NS													
NIG016	40.4	94.9	61.4	84.5	75.2	99.3	99.9	96.1	69.2	85.9	96.6	91.0	82.9
NIG010	28.0	23.5	11.8	17.0	15.1	11.1	70.4	26.1	7.7	6.5	56.1	1.5	22.9
NIG011	36.1	63.9	51.8	43.6	41.5	48.5	47.6	47.4	25.7	26.0	66.6	6.3	42.1
NIGH07	53.2	97.8	87.3	99.2	87.6	59.1	83.3	84.9	99.7	39.8	92.8	53.6	78.2
NIGH09	31.4	52.8	47.2	43.2	58.6	34.8	13.8	16.7	73.6	22.1	12.5	5.9	34.4
NIG017	42.0	80.3	48.2	68.2	70.8	84.7	35.8	63.1	93.0	96.7	40.6	76.2	66.6
NIGH12	74.8	62.3	94.6	56.0	46.3	71.2	75.9	85.0	44.4	82.7	89.8	56.6	70.0
NIG019	78.3	65.5	69.8	54.7	55.4	43.6	81.2	77.0	54.0	63.8	84.9	52.5	65.1
NIGH11	50.9	36.9	16.6	23.9	16.9	40.0	51.9	54.5	15.1	49.5	67.2	11.2	36.2
NIG024	10.9	93.9	91.9	92.3	94.7	76.0	92.5	80.1	98.4	55.5	87.5	66.6	78.4
NIG025	45.4	66.5	77.3	75.7	90.5	65.8	44.9	73.6	56.3	73.6	54.4	87.5	67.6
NIG018	6.9	19.4	19.9	23.8	36.3	12.6	21.5	39.8	32.2	13.0	66.8	7.1	25.0
NIG004	20.9	97.9	62.2	92.9	94.0	96.7	77.7	79.8	96.4	90.1	74.8	56.2	78.3
Average	39.9	65.8	56.9	59.6	60.2	57.2	61.3	63.4	58.9	54.3	68.5	44.0	57.5
UD													
NIG016	47.3	80.9	66.7	91.1	97.4	93.2	40.6	59.4	96.8	74.7	36.9	91.4	73.0
NIG010	26.7	42.5	41.4	30.3	30.9	16.1	73.2	33.7	12.5	12.3	77.7	4.1	33.4
NIG011	29.9	88.4	99.7	92.5	84.5	80.2	67.7	87.4	91.9	49.1	89.8	47.0	75.7
NIGH07	36.8	82.1	74.6	73.9	59.9	93.8	69.6	99.5	50.7	95.1	87.3	65.0	74.0
NIGH09	35.2	84.4	70.3	80.8	63.9	87.0	70.2	63.5	58.3	66.9	50.7	96.4	69.0
NIG017	31.5	33.9	21.3	28.0	14.9	67.4	47.2	48.1	6.6	95.6	42.2	17.4	37.8
NIGH12	92.9	64.1	55.0	55.5	48.4	83.6	53.6	69.5	31.4	96.8	64.2	32.4	62.3
NIG019	61.0	73.2	48.1	50.8	38.4	91.5	52.5	64.4	17.9	96.3	57.9	33.5	57.1
NIGH11	25.1	28.3	15.5	14.3	10.1	27.1	39.2	37.5	5.1	54.5	45.3	2.9	25.4
NIG024	6.1	41.6	29.3	47.0	49.5	42.0	41.4	60.0	42.4	70.8	58.8	26.6	43.0
NIG025	11.7	22.0	15.6	31.4	20.3	93.5	38.7	65.1	17.0	59.5	53.0	17.0	37.1
NIG018	82.5	92.3	68.8	72.2	52.8	75.3	89.7	74.8	63.8	84.9	58.3	43.8	71.6
NIG004	23.1	54.5	31.0	45.5	29.2	85.1	89.0	86.7	40.5	69.4	95.0	57.5	58.9
Average	39.2	60.6	49.0	54.9	46.2	72.0	59.4	65.3	41.1	71.2	62.9	41.1	55.3
